# Internet Use, eHealth Literacy, and Dietary Supplement Use Among Young Adults in Pakistan: Cross-Sectional Study

**DOI:** 10.2196/17014

**Published:** 2020-06-10

**Authors:** Amina Tariq, Shanchita R Khan, Amna Basharat

**Affiliations:** 1 School of Public Health and Social Work Faculty of Health Queensland University of Technology Kelvin Grove Australia; 2 Department of Computer Science National University of Computer and Emerging Sciences Islamabad Pakistan

**Keywords:** internet, digital health, eHealth, eHealth literacy, internet use, physical activity, university students, Pakistan

## Abstract

**Background:**

Increased access to the internet has facilitated widespread availability of health information. Thus, electronic health (eHealth) literacy—the ability to seek, find, understand, and appraise health information from electronic resources and apply that knowledge in making a health-related decision—is a crucial skill. Despite the increasing use of the internet as a source of health information in developing countries, only a few studies have examined the eHealth literacy of young adults, who frequently use the internet to access health information in these developing countries.

**Objective:**

The aim of this study was to assess the patterns of internet use and eHealth literacy levels among university students pursuing a non–health-related degree in Pakistan. We also examined the association of the eHealth literacy levels of these young adults with their physical activity levels and dietary supplement intake.

**Methods:**

Students from 2 leading engineering universities in Pakistan were invited to participate in a cross-sectional anonymous web-based survey in order to collect data on their internet use, eHealth literacy, and dietary supplement intake. Of the 900 eligible university students who were invited to participate, 505 (56.1%) students who completed the questionnaire were included in the analysis. The findings were converted to median values and frequency analyses were performed. The associations between the variables were determined using the chi-square test; *P*≤.05 was considered significant.

**Results:**

In this study, the median eHealth literacy scale (eHEALS) score was 29, which did not vary across gender. The most common type of health-related information that was searched by the participants was that related to maintaining a healthy lifestyle (305/505, 60.4%). Participants with high eHEALS scores were those who used the internet frequently for finding people with similar health issues (*P*<.001). The use of specific social media platforms was not associated with the perceived eHealth literacy levels. Neither the frequency of physical activity nor the dietary supplement use was associated with the eHealth literacy of the participants.

**Conclusions:**

University students in non–health-related disciplines in Pakistan expressed high confidence in their skills to find health-related information on the internet, as indicated by the aggregate eHEALS scores. However, the findings of our study show that the perceived eHealth literacy was not associated with health behaviors such as physical activity and dietary supplement intake. Further research is necessary to investigate the extent to which eHealth literacy can be considered as a panacea for solving public health challenges in developing countries.

## Introduction

The role of internet and smartphone ubiquity in health care improvement, particularly in a resource-constrained developing world, has gained global interest in the past decade [1]. Under the umbrella terms of electronic health (eHealth) and mHealth, researchers have investigated possible ways in which the proliferation of smartphone devices and internet platforms can be used to improve health education and health outcomes in the developing world [2].

In developed countries such as the United States and Japan, more than 70% of the population has reported the use of internet as the primary source of information, especially for self-care management; similarly, in developing countries, the use of internet for accessing health information has been rapidly increasing [3,4]. This trend is not surprising, considering the tech savviness of young adults and the easy accessibility to the internet. However, these web-based health interventions or programs cannot be executed as technical programs in a vacuum, ignoring the complex contexts in which they are implemented [5]. Therefore, in digital health literacy, it is important that consumer-focused electronic resources are designed such that they are aligned with the literacy levels of the intended audience. eHealth literacy is identified as an important skill set for eHealth users, and it refers to the ability of an individual to seek, find, understand, and appraise health information from electronic resources and apply such knowledge to addressing or solving a health problem [6]. The eHealth literacy scale consists of several levels, which show the set of skills that are required to effectively engage the use of information technology for improving health. The lower levels of the scale comprise operational and navigational skills, while the higher levels require the ability to choose and critically evaluate the available information [7]. eHealth literacy can help developing countries access web-based health information resources effectively. Studies examining eHealth literacy [4,8-11] have largely been performed in developed countries such as the United States, Finland, China, and Japan—with particular emphasis on young adults and college students. However, only a few studies have identified an association between eHealth literacy and health behaviors, while even fewer studies have examined the association between eHealth literacy and the health behaviors of young adults in developing countries [12].

Despite the increasing use of the internet as a source of health information in developing countries, eHealth literacy is an unexplored entity, which has been created and refined by the developed world [13]. While technology-based health information is being proposed as the solution to elevate the health literacy levels of the Pakistani population [14], there is very limited investigation of the eHealth literacy of the health seekers—with focus only on those seeking health-related education [15]. The latest National Human Development Report of Pakistan [16] highlighted that only little is known about the health literacy and the health behaviors of Pakistani youths—especially among those who are well-educated and frequent users of the internet.

Therefore, in this study, we aimed to examine the association between the patterns of internet use for seeking health information and the eHealth literacy of university students in Pakistan. Further, we investigated the possible association of eHealth literacy with the health behaviors (physical exercise and dietary supplement intake) of these university students.

## Methods

### Participants

Eligible participants in this study were Pakistani university students aged 18 years or above and they were enrolled in an undergraduate or a postgraduate course primarily in any degree other than in medical or health sciences. The convenience sample of the participants was approached using the university group email lists (N=900) of 2 leading engineering universities in Pakistan by one of the investigators (AB). Participants were expected to have adequate computer skills as they studied in engineering universities with sophisticated technical infrastructure.

### Measurements

A quantitative cross-sectional design was used to conduct this research. Students enrolled in the universities had to meet the English language requirements prior to admission, as the mode of instruction in the anonymous web-based survey was English. Therefore, all the participants had to comprehend and answer the questions in English. The survey instrument was developed based on a review of previous studies consisting of validated items [8,17,18]. The questionnaire consisted of the following key items.

1. Demographic data and patterns of internet use: This item consisted of questions on participant age, gender, and education level. The internet use patterns were explored by asking questions on the frequency of internet use for browsing health information and common health-related topics. These questions were designed on the basis of previous studies exploring the patterns and reasons for seeking web-based health information [17]. Finally, participants rated their frequency of using Google and other social media platforms as a venue for seeking health information on a scale of 1 to 5 (1=Never, 5=Very Often). The participants rated the following social media platforms: Google, WhatsApp, Twitter, Snapchat, Instagram, and Facebook [8].
2. Perceived eHealth literacy: eHealth literacy was measured using the English version of the eHealth literacy scale (eHEALS) [18], which is an 8-item instrument measured on a 5-point Likert-type scale ranging from 1 (strongly disagree) to 5 (strongly agree). The eHEALS scores ranged from 8 to 40, wherein a high score indicates high perceived eHealth literacy and a low score indicates low perceived eHealth literacy.
3. Health behaviors: Physical activity was assessed through questions on the weekly frequency of moderate physical activity. Supplement intake was assessed through questions on the use of dietary supplements. For those who used dietary supplements, further information was requested on the type of supplement used.

The ethical approval for this study was obtained from the Human Research Ethics Committee (Approval number: 1700000897) of Queensland University of Technology. This study has been reported in accordance with the Checklist for Reporting the Results of Internet E-Surveys [19].

### Survey Validation and Administration

A pilot test of the survey was completed with a sample of 14 undergraduate university students in Pakistan to ensure that the target audience understood the meaning of each question and response. The pilot feedback was used to edit the survey accordingly. The revised survey was administered using the web-based Key Survey tool. We believed that a web-based survey would be appropriate for this study because responders to such surveys can use the computer and internet effectively. The identified eligible participants were sent the link to the web-based survey via their university group email and 1 reminder was sent to give them the opportunity to complete the survey if they had not already done so. The participation in this survey was voluntary and no incentives were offered for participation. The survey remained open for 6 weeks (December 2017 to January 2018).

### Data Analysis

A data matrix was produced from the completed questionnaires by using SAS 9.4 for Windows (SAS Institute Inc). Qualitative variables were expressed as numbers and percentages. The percentages were calculated based on the valid cases only. The chi-square test was performed to determine the association between the variables. P≤.05 was considered significant. The total scores of the eHEALS were summed to a range from 8 to 40, with high scores representing high self-perceived eHealth literacy. We divided the eHEALS score into one of the 2 categories (high or low) relative to the median group value (median 29, IQR 26-32); we did this in accordance to that done in previous studies [4,8] that used eHEALS to analyze the associations between the frequency of seeking health information, eHealth literacy, and health behaviors.

## Results

### Participants’ Demographic Data and Internet Use Patterns

Of the 900 eligible students invited to participate, 559 (62.1%) students logged onto the web-based questionnaire platform and 505 (56.1%) students completed the questionnaire by providing all the demographic information; thus, 505 students were included in the final analysis (Table 1). Of these 505 students, 211 (41.8%) students were females; 88.1% (445/505) of the participants were younger than 25 years and 85.3% (431/505) of them were pursuing an undergraduate degree. We found that 79.6% (402/505) of the participants were identified as frequent users of the internet (almost every day); 11.1% (56/505) of the participants reported using the internet for searching health-related information almost every day, while 46.1% (233/505) used it once a week or more.

**Table 1:** Demographic data and internet use patterns of the participants (n=505).

Demographic data and frequency of internet use		Value, n (%)
**Age (years)**		
	16-25	445 (88.1)
	26-30	38 (7.5)
	30+	22 (4.2)
**Gender**		
	Male	294 (58.2)
	Female	211 (41.8)
**Education level**		
	Undergraduate degree	431 (85.3)
	Postgraduate	74 (14.6)
**Frequency of internet use**		
	Almost everyday	402 (79.6)
	Several days a week (≥1 day)	88 (17.5)
	Less than once a week or never	15 (3.0)
**Frequency of internet use for health-related information**		
	Almost everyday	56 (11.1)
	Several days a week (≥1 day)	233 (46.1)
	Less than once a week or never	216 (42.7)

### Internet Use Patterns and eHealth Literacy

With regard to eHealth literacy, only 399 participants who used the internet for finding health-related information answered the 8 standard eHEALS questions. The median eHEALS score was 29 and this value did not vary with gender or the education level. Based on the eHEALS score, the participants were divided into 2 groups: the first group had scores higher than the median eHEALS score (191/399, 47.8%) while the second group had scores lower than the median eHEALS score (208/399, 52.1%). The most common type of health-related information searched by the participants (305/505, 60.4%) was that related to healthy lifestyle (weight, exercise, etc)—this was common among both high and low eHealth literacy groups. However, those with high perceived eHealth literacy reported that they used the internet over the last 12 months more often for finding people with similar health issues (*P*<.001) (Table 2). Google was the most widely used search engine. Of the 401 participants who reported frequent use of Google, 365 (91.0%) used Google to obtain health-related information. With regard to other social media platforms, 64.0% (199/311) of the participants who used Facebook, 59.6% (171/287) of the participants who used Wikipedia, and 15.0% (40/265) of the participants who used Twitter reported that they used these platforms to obtain or share health-related information. The use of any of these specific social media platforms was not associated with the perceived eHealth literacy.

### Health Behaviors and eHealth Literacy

Overall, 25.9% (119/459) of the participants reported engaging in moderate physical activity more than five times a week, while 23.5% (108/459) of the participants engaged in moderate physical activity less than once a week. The perceived level of eHealth literacy was not associated with the reported levels of physical activity (Table 3). Some form of supplement was taken by 48.7% (220/452) of the participants. Vitamin D supplements were reported as the most commonly used supplement (177/413, 42.9%). Dietary supplement use was significantly associated with gender (*P*=.002), with females more likely to use supplements as compared to males. The perceived level of eHealth literacy was not associated with the reported supplement use patterns (Table 3).

**Table 2:** Association between internet use patterns and eHealth literacy (n=399).

Internet use patterns and eHealth literacy			Value, n (%)			*P* value
			eHEALS^a^ Low	eHEALS High		
**Frequency of internet use for health-related information**						.02
		Several days a week or more	62 (44.3)	78 (55.7)		
		Once a week or less	146 (56.4)	113 (43.6)		
**Types of health-related information searched on the internet**						
		Healthy lifestyle	149 (50.7)	145 (49.3)	.33	
		Medication	69 (52.3)	63 (47.7)	.97	
		About a particular disease	84 (51.5)	79 (48.5)	.84	
		Find a care provider or hospital	26 (52.0)	24 (48.0)	.98	
		Peer support forums/other	12 (26.7)	29 (73.3)	.004	
**Frequency of using internet over the last 12 months for finding people with similar health issues**						<.001
		Often	43 (20.7)	76 (39.8)		
		Rarely (less than once a month)	124 (59.7)	93 (48.7)		
		Never	41 (19.7)	22 (11.5)		
**Social media platforms used to obtain/share health information or discuss health problems**						
	**Google**					.91
		No	17 (51.5)	16 (48.5)		
		Yes	187 (52.5)	169 (47.5)		
	**Facebook**					.15
		No	57 (53.8)	49 (46.2)		
		Yes	87 (45.1)	106 (54.9)		
	**Twitter**					.74
		No	106 (49.1)	110 (50.9)		
		Yes	18 (46.2)	21 (53.8)		
	**Wikipedia**					.54
		No	57 (51.4)	54 (48.6)		
		Yes	79 (47.6)	87 (52.4)		
	**WhatsApp**					.60
		No	81 (50.6)	79 (49.4)		
		Yes	56 (47.5)	62 (52.5)		
	**Others (eg, Reddit)**					.51
		No	63 (50.8)	61 (49.2)		
		Yes	25 (45.5)	30 (54.5)		

^a^eHEALS: eHealth literacy scale.

**Table 3:** Association between eHealth literacy and health behaviors.

Health behaviors			Value, n (%)				*P* value
			eHEALS^a^ Low		eHEALS High		
**Frequency of moderate physical activity (30-min activity)**							.85
	More than 5 times a week	54 (52.9)		48 (47.1)			
	1-4 times a week	91 (49.7)		92 (50.3)			
	Less than once a week	46 (52.3)		42 (47.7)			
**Dietary supplement intake (Vitamin D and others)**							.25
	No	99 (54.4)		83 (45.6)			
	Yes	93 (48.4)		99 (51.6)			
**Types of supplements**							
	Vitamin D	74 (47.1)		83 (52.9)		.14	
	Calcium	26 (59.1)		18 (40.9)		.33	
	Vitamin C	25 (62.5)		15 (37.5)		.17	
	Vitamin B complex	12 (48.0)		13 (52.0)		.67	
	Iron	21 (63.6)		12 (36.4)		.17	
	Mineral supplements (zinc, magnesium, etc)	11 (52.4)		10 (47.6)		.98	
	Multivitamins (not containing vitamin D)	8 (42.1)		11 (57.9)		.37	

^a^eHEALS: eHealth literacy scale.

## Discussion

To our knowledge, this study is one of the very few studies that have examined the eHealth literacy levels among young adults in developing countries and the association of eHealth literacy with internet use patterns and health behaviors. After controlling for the sociodemographic variables, we found that eHealth literacy was not associated with health behaviors such as physical activity and dietary supplement intake. Although there is evidence that eHEALS may consist of several subscales, we analyzed the eHEALS as a unidimensional factor, following Hyde’s recommendation [20]. Therefore, our analysis considers the full eHEALS, which also allows us to compare our results with those of other studies [8].

Our population consisted of university students pursuing an engineering degree in Pakistan; the median eHEALS score in our study was similar to that reported among young adults seeking a health-related degree in Pakistan [15]. Further, the median eHEALS score in this study was comparable to that reported previously in an adult population in the United States (mean eHEALS score, 29.0), slightly higher than that reported in the adult populations of Korea, Iran, and Kuwait (mean eHEALS scores, 28.0, 28.2, and 28.6, respectively), and much higher than that reported in an adult population in Japan (mean eHEALS score, 23.4) [4,8-10]. Similar to that reported in previous studies, most sociodemographic variables (eg, gender, age) examined in this study were not significant predictors of eHealth literacy [4,8-10]. As reported previously [4,10], it is possible that the geographical location and cultural and language barriers could affect the eHEALS scores because of limited availability of health-related information in languages other than English. This may not be the case for the population in this study as English is the official medium of instruction in the Pakistani university educational system and participants in this study are frequent users of the internet.

Unlike the findings of recent studies from the United States and Japan [4,21] that showed a significant association between eHEALS scores in young adults and health behaviors such as exercise, we found no association between eHEALS scores and the reported physical activity levels. In Pakistan, very few studies have investigated how digital health literacy can improve health behaviors. In 2018, Saeed et al [22] evaluated the digital health literacy of patients with type 2 diabetes (N=204) in Lahore, Pakistan, and they reported that patients with high socioeconomic status agreed that access to digital medical content via smartphones and tablets helped them improve their health. However, only the use of internet and the high eHealth literacy levels among educated young adults cannot translate into health behavior improvements (eg, increase in the physical activity levels) if the communities that they live in lack the needed infrastructure to facilitate the required physical activity programs. It is important to note that developing countries such as Pakistan—unlike Japan and the United States—lack culturally suitable infrastructure (eg, safe outdoor facilities for young women) and civic amenities such as playgrounds, parks, and gyms (even within universities) [23,24]. The lack of such facilities inhibits the realization of positive health behaviors and encourages indoor sedentary lifestyles [16].

We also found that there was no significant association (*P*=.25) between eHealth literacy and dietary supplement intake. Recent studies from developed countries have reported internet as the primary source of information to inform attitudes toward the use of dietary supplements among young adults [25,26]. Recent evidences have shown that eHealth literacy influences the food choices of adolescents [27]. In the last few years, Pakistan has witnessed a rise in the demand for nutraceuticals; dietary supplements attained a compound annual growth rate of 14% in 2016 [28]. These nutraceuticals are mostly used by the educated population [29]. In light of these interesting preliminary trends, future research should focus on the effects of the patterns of internet searches and social behaviors on supplement intake. The aspects around eHealth literacy and its association with health behaviors such as dietary supplement use have not been reported in any developing country to date, to the best of our knowledge. This study presents a valuable contribution to establish evidence, in particular, for a country facing pressures to improve health literacy and public health outcomes.

This study has several limitations. First, the participants were recruited from a convenience sample of university students in Pakistan; thus, the relationships assessed may have been biased because of the potentially non-representative nature of this sample. However, this approach was viable because it provided us with a sizable sample in a reasonable amount of time for a population wherein such investigations have not been performed previously. Our study presents the needed ground to build on future studies that explore the eHealth literacy of broad population groups so that the findings can be applied to other developing countries. Second, as in previous studies, health behavior and eHealth literacy were examined using a self-administered questionnaire. Thus, inaccuracies in estimating the health behavior and eHealth literacy level were unavoidable, and data of some of the participants were missing. In addition, we did not investigate the specific health status of the participants or whether they were searching for health-related information for another family member. Moreover, this study was limited to only examining supplement use as a dietary behavior. Future studies should explore additional dietary behaviors (eg, nutritional intake, smoking) and the context for using the internet for health-related information. Our findings revealed high eHEALS scores among the participants. This high confidence shown by the university students presents a myriad of opportunities to better engage people digitally and conveniently. As internet is being increasingly used as a source of health information, further research is needed to identify the mechanisms linking eHealth literacy with health behaviors toward designing contextually effective strategies for improving self-care in developing countries.

## Abbreviations

eHEALS: eHealth literacy scale
eHealth: electronic health
